# The α2‐Subunit Preferring GABA Positive Allosteric Modulator, N‐Desmethyl‐Clobazam in Peripheral Neuropathic Pain Patients: Results of a Phase IIa Randomised Placebo‐Controlled Ascending Dose Study

**DOI:** 10.1002/ejp.70413

**Published:** 2026-10-16

**Authors:** M. Besson, A. Matthey, Y. Daali, A. Ramsahye, C. Samer, J. Desmeules, F. Curtin

**Affiliations:** ^1^ Division of Clinical Pharmacology and Toxicology, Department of Acute Care Medicine Geneva University Hospitals Geneva Switzerland; ^2^ Department of Anesthesiology, Pharmacology, Intensive Care and Emergency Medicine, Faculty of Medicine University of Geneva Geneva Switzerland; ^3^ Institute of Pharmaceutical Sciences of Western Switzerland (ISPSO) University of Geneva Geneva Switzerland

## Abstract

**Background:**

N‐desmethyl clobazam (NDMC), an α2‐preferring‐α1‐sparing‐GABA_A_ modulator, was promising in preclinical models and Phase I studies, demonstrating antihyperalgesic properties without associated sedation. This Phase IIa trial aimed at exploring the effect of NDMC in peripheral neuropathic pain (NP) patients.

**Method:**

Randomised, ascending doses, double‐blind, placebo‐controlled study, evaluating 40, 60, 120 mg of NDMC. Primary outcome was the change in weekly average daily pain (WADP) on a Numerical Rating Scale (NRS) from baseline (V1) to the end of the study (V7). Secondary outcomes included sedation (subjective NRS), quality of life (SF36), global and patient impression of change (CGI, PCGI). PK and safety data were collected. The study was prematurely terminated due to recruitment challenges related to the COVID‐19 pandemic.

**Results:**

Thirty‐three out of the planned 76 patients were enrolled. The mean reduction in WADP from V1 to V7 was −1.53 points for placebo and −1.99 across all NDMC doses (delta = 0.46, *p* = 0.47). When analysed according to NDMC plasma concentrations, the mean WADP reduction was 1.53 (SD 1.58) for placebo 1.78 (SD 1.79) for NDMC < 1000 ng/mL, and 2.40 (SD 1.46) for NDMC ≥ 1000 ng/mL. The prevalence of 30% pain reduction was 38.5% (placebo) and 83.3% (NDMC ≥ 1000 ng/mL). Logistic regression analysis indicated that NDMC ≥ 1000 ng/nL was predictive of a 30% pain reduction (odds ratio = 12.5, *p* = 0.04). NDMC was well tolerated.

**Conclusion:**

Despite being underpowered, this study revealed a consistent exposure–response relationship along with a favourable safety profile for NDMC in patients with NP.

**Trial Registration:**

ClinicalTrial.gov (NCT03375034); kofam.ch (SNCTP000003517).

**Significance Statement:**

This work presenting the Phase II data of the new antihyperalgesic compound, N‐Desmethyl‐Clobazam (NDMC) in peripheral neuropathic pain patients support further investigation into the role in modulating GABAergic pathways as a potential therapeutic target for neuropathic pain. This objective is particularly relevant given the limited efficacy of standard pharmacological treatments for neuropathic pain, which often requires a multimodal approach targeting the multiple pathways involved in pain pathophysiology.

## 1 Introduction

Neuropathic pain (NP), defined as pain resulting from damage to or disease of the somatosensory nervous system (IASP 2026), is a therapeutic challenge requiring a multimodal and integrative approach. Pharmacological treatment has seen minimal progress over the past two decades, and its effectiveness continues to be limited (Soliman et al. 2025). Part of this is related to adverse effects such as sedation, limiting the use of analgesics in daily practice. From a pharmacological perspective, the trend has always been towards identifying analgesic targets based on the mechanisms of underlying neuropathic pain (Zeng et al. 2025).

Dysfunction of synaptic inhibition within the spinal cord is one of the contributing factors to central sensitisation that governs NP (Zeilhofer 2005, 2008). Hence, facilitation of GABAergic inhibition in the dorsal horn through GABAA receptors allosteric modulation is a rational approach to compensate; nevertheless, it is potentially limited by an associated sedation (Zeilhofer et al. 2012). Benzodiazepine‐sensitive GABAA receptors contain two identical *α* subunits (α1, α2, α3 or α5), two β subunits and one γ2 sub‐unit (Zeilhofer et al. 2009). Studies in genetically modified mice showed that compounds targeting the α1 sub‐unit containing GABAA receptor primarily induce sedation, whereas compounds with greater selectivity for α2 and α3 subunits exhibit antihyperalgesic properties (Di Lio et al. 2011; Knabl et al. 2008; Ralvenius et al. 2015). However, all identified selective compounds failed early in clinical development (Rudolph and Knoflach 2011) because they all were only partial agonists at α‐2 receptors and lost their selectivity at doses required for analgesia in humans (van Amerongen et al. 2019; Gurrell et al. 2018; Ralvenius et al. 2015).

We previously demonstrated that the 1,5‐benzodiazepine clobazam (CBZ), used in epilepsy for decades and reported for less cognitive effect than other benzodiazepines, was effective in reducing hyperalgesia in animal and human experimental models of central sensitisation (Besson et al. 2013, 2015). Further in vitro and in vivo investigations using recombinant receptor models evidenced that N‐Desmethyl‐Clobazam (NDMC), CBZ's main metabolite, demonstrated a clear preference for α2 over α1 subunits across a wide concentration range and a higher efficacy on α2‐subtype receptors than diazepam (Ralvenius et al. 2016).

Hence, we studied NDMC in a Phase I, randomised, controlled trial using an experimental UVB‐induced pain model in healthy subjects (Besson et al. 2015).

Measured NDMC plasma concentrations after single doses were lower than expected, precluding proper pharmacodynamic assessments. Still, NDMC had linear pharmacokinetics with a substantial five times drug accumulation with repeated daily administrations. NDMC was free of sedation at any of the doses tested, including repeated daily administrations (Matthey et al. 2020). These properties made NDMC the first in class GABAergic allosteric modulator possessing all attributes for producing adequate analgesia in NP patients with an optimised safety. Indeed, due to its metabolic filiation to the registered drug CBZ, this compound already benefits from an over 40 years documented safety profile.

We present here the results of a Phase IIa randomised placebo‐controlled ascending dose study of NDMC in patients with neuropathic pain.

## 2 Material and Methods

The study took place at Geneva University Hospitals (HUG) from June 2020 (first patient in) to September 2022 (last patient out).

The study was conducted in accordance with the principles enunciated in the Declaration of Helsinki and as outlined in the International Council on Harmonisation of Technical Requirements for Registration of Pharmaceuticals for Human Use (ICH) Guidelines E6 (Good Clinical Practice: Consolidated Guidance) and Swiss federal regulations. The protocol and informed consent were reviewed by the local independent Ethics Committee and the Swiss agency for pharmaceutical products, Swissmedic. The trial was registered within ClinicalTrial.gov (NCT03375034) and within kofam.ch (SNCTP000003517).

The study was supported by public funding only (Swiss innovation agency (Innosuisse) Grant 37848.1 IP‐LS). The rules governing this fund stipulated that the study had to be completed within a specified timeframe. However, the study took place during the SARS Cov‐2 pandemic, which had a decisively broad negative impact on patient's recruitment and conduct of the study. The initial exceptional extension of the timeframe proved insufficient to complete the study as planned. An interim analysis was therefore performed to ensure compliance with the funding agency, after one third of the planned recruitment for Cohort 3, marking the completion of an initial stage and providing exploratory pharmacokinetic and pharmacodynamic data. This approach was consistent with the framework of a Phase IIa study.

### 2.1 Study Design and Treatment

The study was a randomised, double‐blind, placebo‐controlled, 4 ascending dose arms, sequential parallel study with the following treatment groups: NDMC 40 mg/day (6 patients), NDMC 60 mg/day (6 patients), NDMC 120 mg/day (28 planned patients) and placebo (36 planned patients). The doses were chosen according to our preclinical and pharmacokinetic data (Matthey et al. 2020; Ralvenius et al. 2016) (for details, see Supporting Information). Moreover, 20 and 40 mg were also tested for safety purposes.

The randomisation ratio was NDMC40 : NDMC60: NDMC120 : Placebo 1:1:5:5. Each cohort followed its own randomisation list. The allocation to the treatment groups was computer‐generated (random sized blocks) by the Methodology Support Unit of the Clinical Research Center of the Geneva university hospital. Investigators and patients were blinded.

The 3 cohorts were separated by two interim analyses to check for safety and PK linearity of the repeated dosing of NDMC under the supervision of an unblinded Data Safety Monitoring Board (DSMB). Each cohort included an active and a placebo arm. Patients were followed up for 8 weeks (2 weeks of titration, 4 weeks of maintenance dose and 2 weeks of down titration and wash‐out).

N‐desmethylclobazam (NDMC), which is chemically known as 8‐Chloro‐1‐phenyl‐1H‐1,5‐benzodiazepine‐2,4(3H,5H)‐dione, came as a 20 mg capsule and was administered per os. Placebo capsules were manufactured from the same excipients (inactive substances) found in the galenic formulation of the active capsules.

### 2.2 Patients

Male and female patients, between 18 and 65 years, were included after having signed an informed consent if they had a diagnosis of small fibre neuropathy, diabetic peripheral neuropathy, post‐herpetic neuralgia, HIV‐associated neuropathic pain, post‐traumatic/postoperative peripheral neuropathy, chemotherapy‐associated peripheral neuropathy or radicular low back pain with sensory deficit. The minimal score on the DN4 questionnaire (Bouhassira et al. 2005) was 4. Pain duration was required to be at least 3 months and the preceding week average daily intensity pain score was at least 4 on a numerical rating scale (NRS).

In addition, an insufficient response to at least one attempt with one of the currently recommended pharmacological treatments for neuropathic pain taken at recommended dose, or a failure of such a treatment because of adverse effect, or a previous refusal of pharmacological pain treatment was required. For patients on concomitant pain medication (antidepressant, gabapentinoids and others) whose pain condition was not controlled, the dose had to remain stable throughout the study.

Patients were excluded if they had any contraindications to benzodiazepines, a history of drug or alcohol abuse in the past 5 years, a current unstable psychiatric disorder or epilepsy.

Female patients were excluded if they were pregnant or breast feeding or intended to become pregnant and were asked to use a highly effective method of contraception. Changes in existing concomitant non‐pharmacological pain management interventions were not permitted throughout the study.

### 2.3 Efficacy Assessments

#### 2.3.1 Primary Outcome

##### 2.3.1.1 Weekly Average of Daily Pain Intensity Score on a Numerical Rating Scale (NRS)

Patients were instructed to self‐rate their average daily pain once every day in an electronic diary.

The weekly average of daily pain (WADP) intensity score was derived from the 7 (at least 5) recordings preceding the visits. NRS was 0 “no pain” to 10 “worst possible pain”. The primary endpoint was the change from baseline to final evaluation (Week 8) in the arithmetic mean of the last 7 (at least 5) entries in the diary.

#### 2.3.2 Secondary Outcomes

##### 2.3.2.1 Weekly Average of Daily Subjective Feeling of Sedation on a Numerating Rating Scale (NRS)

Patients were instructed to self‐rate once every day their average daily somnolence in an electronic diary. The weekly average of daily sedation (WADS) feeling score was derived from the 7 (at least 5) recordings preceding the visits. NRS was 0 “not feeling sedated” to 10 “feeling highly sedated”. The endpoint was the change from baseline to final evaluation (Week 8) in the arithmetic mean of the last 7 (at least 5) entries in the diary.

##### 2.3.2.2 Global Impression of Change as Measured by the Clinical Global Impression (CGI)

The CGI is a 7‐point scale: from 1 (very much improved), 2 (much improved), 3 (minimally improved), 4 (no change), 5 (minimally worse), 6 (much worse) to 7 (very much worse). The investigator rates the patient by answering the following question: “Please, indicate how much the patient's condition has improved or worsened relative to a baseline state at the beginning of the intervention”. The endpoint was the change from baseline to final evaluation (Week 8) in the arithmetic mean of the CGI questionnaire score.

##### 2.3.2.3 Patient Global Impression of Change (PGIC)

The PGIC questionnaire is a 7‐point scale: from 1 (very much improved), 2 (much improved), 3 (minimally improved), 4 (no change), 5 (minimally worse), 6 (much worse) to 7 (very much worse). The patient answers the question: “Please, indicate how you feel now, compared to how you felt before receiving treatment in this study”. The endpoint was the change from baseline to final evaluation (Week 8) in the arithmetic mean of the PGIC questionnaire score.

##### 2.3.2.4 Short Form Questionnaire (SF‐36)

The SF‐36 questionnaire is a health status questionnaire consisting of 36 interview questions. The SF‐36 has eight scaled scores, assessing dimensions such as health, physical, emotional and social functioning; the scores are weighted sums of the questions in each section and range from 0 (more disability) to 100 (less disability). The endpoint was the change from baseline to final evaluation (Week 8) in the means for the eight SF‐36 scores.

### 2.4 Safety Assessments

Safety assessment was based on spontaneously reported or observed treatment emergent adverse events (TEAE). Severity and relationship to the study treatments were determined by the investigator. All TEAEs reported from the time of study drugs administration were collected. Premature discontinuation was recorded.

### 2.5 Pharmacokinetics

Blood samples were collected in 3‐mL EDTA hemogard tubes. Plasma was stored at a temperature below −20°C until analysis. NDMC plasma concentrations were measured using a previously described LC–MS/MS method (Vuilleumier et al. 2013). Each evaluation included a group of six participants, and samples were collected at visit 5 and visit 7 for all subjects before drug administration. NDMC steady‐state concentration (*C*_ss_) was assessed following repeated administration of NDMC, with measurements at Week 5 and Week 7.

### 2.6 Statistical Analysis

The computation of the sample size was based on a one‐sided test of difference of efficacy between the 120 mg NDMC dose group and placebo. The difference in the change from baseline on the NRS pain scale between groups was expected to be 1.5 points with a standard deviation of 2.0 (equivalent to an effect size of 0.75). The power was set at 80% and the two‐sided *α*‐level was 5%. These assumptions required a group size of 22 patients for NDMC 120 mg and placebo groups. Assuming an attrition rate of 30%, at least 32 patients per group were planned. Patients were randomised in each cohort as follows: Cohort 1: NDMC 40 mg *n* = 6, Placebo *n* = 2; Cohort 2: NDMC 60 mg *n* = 6, Placebo *n* = 2; Cohort 3: NDMC 120 mg *n* = 32, placebo *n* = 28; the total sample size was planned to be 76.

Primary efficacy per treatment group was sequentially tested according to the 3 treatment cohorts. A sequential analysis was performed to maintain the overall *α*‐level of 5%: (1) NDMC maximal dose (120 mg/day) vs. placebo, (2) if significant then NDMC 60 mg/day vs. placebo (3) if significant then NDMC 40 mg/day vs. placebo (Wiens 2003).

Post hoc analyses were performed according to three groups based on serum NDMC concentration: groups with NDMC concentration of 0 μg/L, group with NDMC serum concentrations below 1000 μg/L, group with NDMC serum concentrations ≥ 1000 μg/L. The cut‐off was based on in vitro experiments suggesting that serum concentrations above 855 μg/L would allow to reach a CNS concentrations of NDMC in the range of the EC50 values to potentiate the α2‐GABAa receptor within the CNS (Laux and Koeppen 1984; Ralvenius et al. 2016), which was confirmed by observations of the NDMC efficacious concentrations in refractory epilepsies (Haigh et al. 1987; de Leon et al. 2013). To test trends related to concentrations, values of NRS pain, NRS somnolence, score of SF36 scales, CGI, PGIC were analysed by linear regression and percentage of responders, defined by patients improving their NRS pain score by 30% or more, were analysed by logistic regression.

Spearman correlation coefficient was used to assess the linear relationship between NDMC doses and serum concentrations. The tolerability/safety data were evaluated descriptively. Safety data were evaluated by standard summary statistics and additional descriptive methods. Missing data were replaced by a last observation carried forward (LOCF) method.

## 3 Results

### 3.1 Subject Disposition and Baseline Characteristics

For the reasons outlined in the method section, only 60 male and female patients were screened for the study. Thirty‐three were randomised and received at least one dose of the treatment (ITT population). Among them, 30 received all the treatment and completed the study; three patients discontinued. The patient disposition is summarised in Figure 1.

**FIGURE 1:**
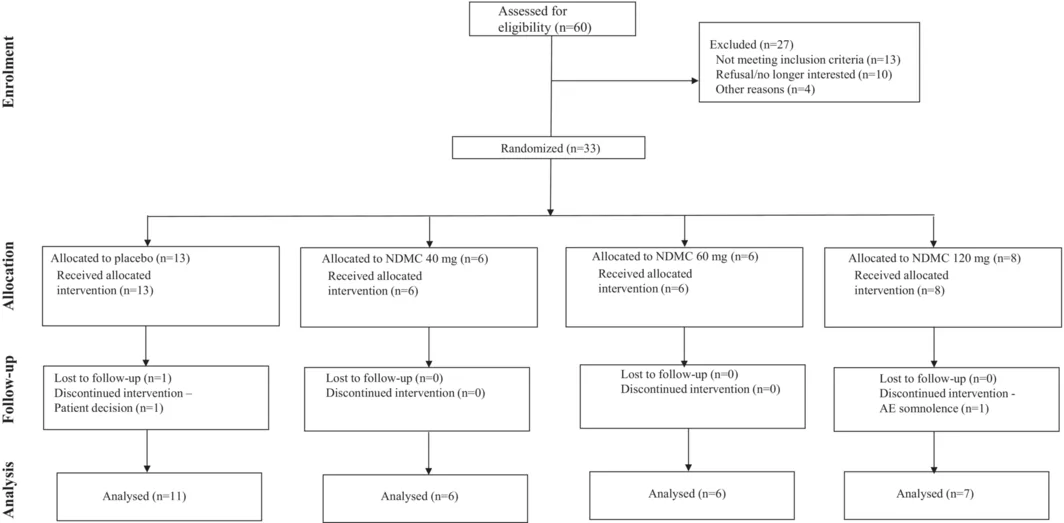
Patient disposition in the randomised control study.

Patients' characteristics are displayed in Table 1.

**TABLE 1:** Patient baseline characteristics by treatment groups.

		Placebo *n* = 13	NDMC40 *n* = 6	NDMC60 *n* = 6	NDMC120 *n* = 8
Age, years mean (SD)		63.4 (9.8)	49.2 (7.7)	61.3 (9.8)	59.8 (17.7)
Sex, Female(F)/Male(M)		5F/8M	4F/2M	6F/0M	7F/1M
Weight, kg mean (SD)		71.6 (16.6)	74.0 (18.1)	68.2 (12.5)	74.0 (15.7)
Height, cm mean (SD)		170.0 (6.3)	169.8 (11.0)	165.5 (7.3)	164.3 (8.4)
Duration, years mean (SD)		7.3 (6.6)	3.2 (3.3)	3.3 (3.1)	8.2 (12.2)
Neuropathic pain cause	Diabetic PNP	1	0	1	0
	Post‐herpetic neuralgia	2	0	0	1
	Post‐chimiotherapy PNP	0	0	2	1
	Post‐surgery neuropathy	2	2	1	3
	Lumbar radiculopathy	4	1	1	1
	Small fibre PNP	3	2	1	1
	Other	1	1	0	1
Concomitant analgesic treatment	Gabapentinoids^a^	3	1	1	0
	Antidepressants^a^	3	2	1	2
	Other antalgic^a^	5	4	3	1
	None	4	2	2	5

Abbreviation: PNP, polyneuropathy.

^a^ Some patients had more than one concomitant pain medication.

The mean age of the cohort was 59.5 years (SD 12.5) and was similar across groups apart from group NDMC 40 mg wherein patients were slightly younger. Two thirds of patients were female. The duration of the neuropathic pain was between 3 and 8 years and was longer in the placebo and NDMC 120 mg groups. Neuropathic pain from post‐surgical origin, radicular low back pain and small fibre neuropathy were the most frequent causes of chronic neuropathic pain, and these different causes were distributed in a rather balanced way across groups. Antidepressants were the most frequent type of analgesic concomitant medication, followed by gabapentinoids. Other treatment included anti‐inflammatory drugs or opioids. About 40% of patients had no concomitant pain medication when starting the clinical trial.

### 3.2 Efficacy

#### 3.2.1 Primary Endpoint Analysis Per Treatment Groups

Table 2 presents the analysis of the primary endpoint, which was the difference of weekly average of daily pain intensity score on a Numerical Rating Scale (WADP NRS) between baseline and final evaluation (after 4 weeks of maintenance dose).

**TABLE 2:** Difference in the weekly average of daily pain intensity (wadp) score measured on a numerating rating scale (NRS) between baseline and final evaluation (4 weeks of treatment maintenance) on ITT population defined on patients having completed the whole study, by treatment group (LOCF when missing).

	Placebo *n* = 11		NDMC 40 mg *n* = 6		NDMC 60 mg *n* = 6		NDMC 120 mg *n* = 7		All NDMC *n* = 19	
	Mean	SD	Mean	SD	Mean	SD	Mean	SD	Mean	SD
	−1.53	1.58	−2.45	2.10	−1.80	1.41	−1.67	1.49	−1.96	1.62
Sequential testing NDMC doses vs. placebo			*t*‐test vs. placebo −1.03 *p* = 0.19		*t*‐test vs. placebo −0.35 *p* = 0.95		*t*‐test vs. placebo −0.19 *p* = 0.85			
*t*‐test all NDMC doses vs. placebo									*t*‐test vs. placebo −0.71 *p* = 0.49	

A decrease in the WADP NRS scores was observed in all treatment groups; the mean WADP NRS score (SD) decreased by 1.5 points (1.6) in the placebo group compared to a decrease of 2.5 points (2.1) in the NDMC 40 mg group, 1.8 points (1.4) in the NDMC 60 mg group and of 1.7 points (1.6) in the NDMC 120 mg group. None of the sequential *t*‐tests showed a statistically significant difference between any NDMC dose groups and the placebo group. By pooling all patients receiving NDMC, the decrease in the WADP NRS pain score was of 2 points (1.6) in NDMC‐treated patients versus 1.5 in the placebo group (1.6); the difference (0.43) was not statistically significant (*t*‐test value −0.71, *p* = 0.49).

#### 3.2.2 Efficacy According to NDMC Concentrations

Table 3 presents the post hoc analysis results of the responder rate and the difference in WADP NRS pain score according to NDMC plasma concentrations.

**TABLE 3:** Difference in the weekly average of daily pain intensity (wadp) score measured on a numerating rating scale (NRS) between baseline and final evaluation (4 weeks of treatment maintenance) and responder rate, defined as a > 30% decrease in the wadp NRS score, according to NDMC plasma concentrations; post hoc analysis.

	NDMC conc. = 0 ng/mL, *n* = 11		NDMC conc. < 1000 ng/mL, *n* = 13		NDMC conc. ≥ 1000 ng/mL, *n* = 6		Linear reg. *p*
	Mean	SD	Mean	SD	Mean	SD	
Change in NRS pain score	−1.53	1.58	−1.75	1.71	−2.40	1.46	0.31
	*n*	%	*n*	%	*n*	%	Logistic reg. *p*
Responder (decrease of > 30% in NRS pain)	5	45%	4	31%	5	83%	0.04

With respect to the relationship between dose and drug exposure, in the 40 mg, no one has a concentration higher than 1000 μg/L; in the 60 mg group, one patient out of 6 had a concentration higher than 1000, and in the 120 mg group, 5 patients out of 6 had a concentration higher than 1000, whereas the reminder had a concentration below. The patient having a concentration > 1000 in the 60 mg group was a poor metaboliser for CYP2C19, which is NDMC main metabolite pathway. The patient having a low concentration in the 120 mg group had a normal phenotype for CYP2C19, and we suspected an observance issue.

The percentage of responders was higher in the ≥ 1000 ng/mL NDMC plasma concentration group with 5 out of 6 (83%) patients being responders whereas in the placebo or < 1000 ng/mL NDMC plasma concentration groups, the responder rates were 45% and 31% respectively. By logistic regression, the odds ratio of being a responder was 12.5 (95% CI: 1.1 to 143.5) in the > 1000 ng/mL NDMC group (*p* = 0.04).

Figure 2 shows the box plots of the WADP NRS scores at baseline and at final evaluation (after 4 weeks treatment maintenance). The WADP NRS median score was 5.3 in the placebo group and 6.9 in > 1000 ng/mL NDMC plasma concentration group at baseline suggesting a higher severity in the latter. After 4 weeks of treatment maintenance, the median WADP NRS scores had all decreased to around 4.0 points in the three concentration groups. This translated into a trend for a higher reduction of the WADP NRS score depending on NDMC plasma concentrations, with a mean reduction (+/−SD) of 1.5 (1.6) points in the placebo group and of 1.8 (1.8) and 2.4 (1.5) points in the < 1000 ng/mL and the ≥ 1000 ng/mL NDMC plasma concentration groups, respectively. This trend was nevertheless not statistically significant (linear regression *p* = 0.31, see Table 3).

**FIGURE 2:**
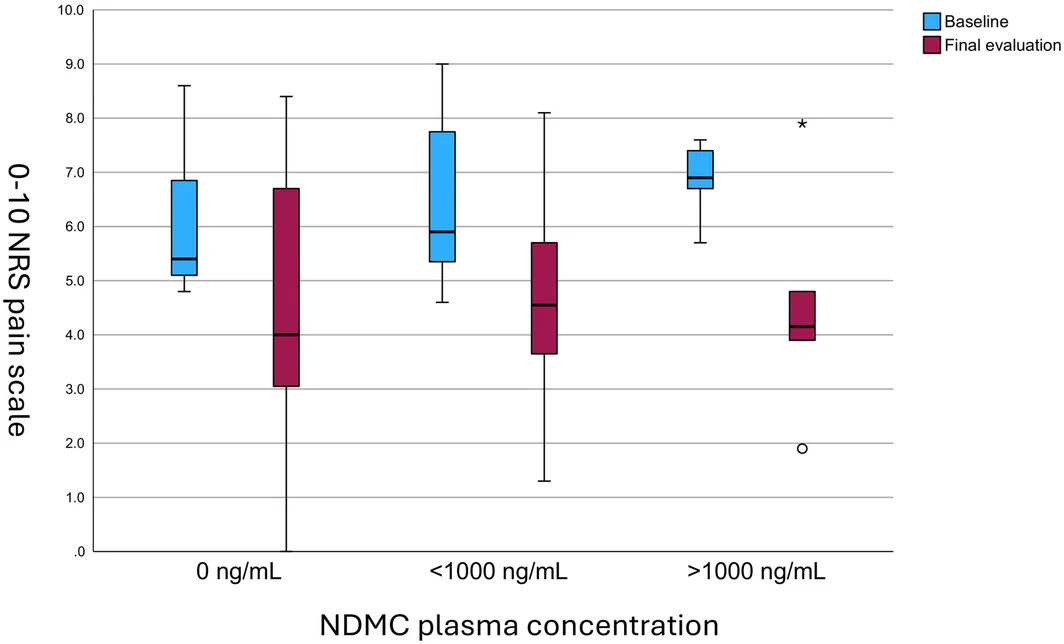
Box plots (min, Q25, median, Q75, max) of Weekly average of daily pain intensity (WADP) score on a 0 to 10 numerical rating scale (NRS) (mean +/− SD) at baseline and at final evaluation according to NDMC plasma concentrations. Post hoc analysis; final evaluation took place after 4 weeks of treatment maintenance.

#### 3.2.3 Clinical Global Impression of Change and Quality of Life According to NDMC Plasma Concentration

Table 4 presents the data of post hoc analysis results for the global impression of change scores and the quality of life according to the NDMC plasma concentration groups.

**TABLE 4:** Difference in global impression of change and SF36 scores between baseline and final evaluation (4 weeks of treatment maintenance) according to NDMC plasma concentrations; post hoc analysis.

		NDMC conc. = 0 ng/mL, *n* = 11		NDMC conc. < 1000 ng/mL, *n* = 13		NDMC conc. ≥ 1000 ng/mL, *n* = 6		Linear reg. *p*
		mean	SD	mean	SD	mean	SD	
Global impression	PGIC	3.36	0.81	3.33	0.78	2.67	1.37	0.18
	CGI	3.09	1.04	2.75	0.87	2.67	1.21	0.36
SF36 Quality of life	Physical functioning	1.0	12.0	5.8	12.9	0.0	6.3	0.97
	Role‐physical	0.5	19.2	7.7	23.5	5.0	20.0	0.61
	Role—emotional	15.0	41.2	1.9	36.0	16.7	30.3	0.93
	Vitality	−13.3	32.2	−12.8	53.6	11.1	27.2	0.32
	Mental health	5.0	18.8	4.8	16.6	27.1	20.0	0.05
	Social functioning	9.2	10.6	10.9	8.6	15.5	24.7	0.40
	Bodily pain	−3.0	17.4	5.4	15.6	13.3	10.8	0.04
	General health	1.2	9.3	2.8	14.4	16.7	12.0	0.04

Both clinical global impression PGIC (from patient point of view) and CGI (from investigator point of view) scores were not different across groups although a slight but non‐statistically significant decrease was observed for both scores in the ≥ 1000 ng/mL NDMC plasma concentration group.

In terms of quality of life, changes from baseline in several dimensions of the SF36 differed between the groups for dimensions such as mental health, bodily pain and general health. The change in the scores suggested an improvement in the ≥ 1000 ng/mL NDMC plasma concentration groups compared to < 1000 ng/mL and the placebo groups and were statistically significant. That was not the case; this was the case for physical functioning, limitation due to physical health, limitation due to emotion, fatigue and social functioning.

### 3.3 Somnolence and Global Safety

Figure 3 and Table 5 detail the weekly average of daily somnolence score on a Numerical Rating Scale (WADS NRS) at baseline and final evaluation according to the NDMC plasma concentration groups. WADS NRS at baseline was heterogenous among groups and an increase in somnolence was reported in all treatment groups at final evaluation (after 4 weeks of treatment maintenance). A trend towards increased somnolence with rising concentrations was observed. Mean scores (+/−SD) were from plus 0.2 (1.4) points in the placebo group, to plus 0.5 (1.0) and 1.3 (1.7) points in the < 1000 ng/mL and the ≥ 1000 ng/mL NDMC plasma concentration groups, respectively. This trend did not reach statistical significance (linear regression *p* = 0.12, Table 4). Of note the final WADS NRS score in the ≥ 1000 ng/mL NDMC plasma concentration group was still within the range of the WADS NRS scores observed at baseline.

**FIGURE 3:**
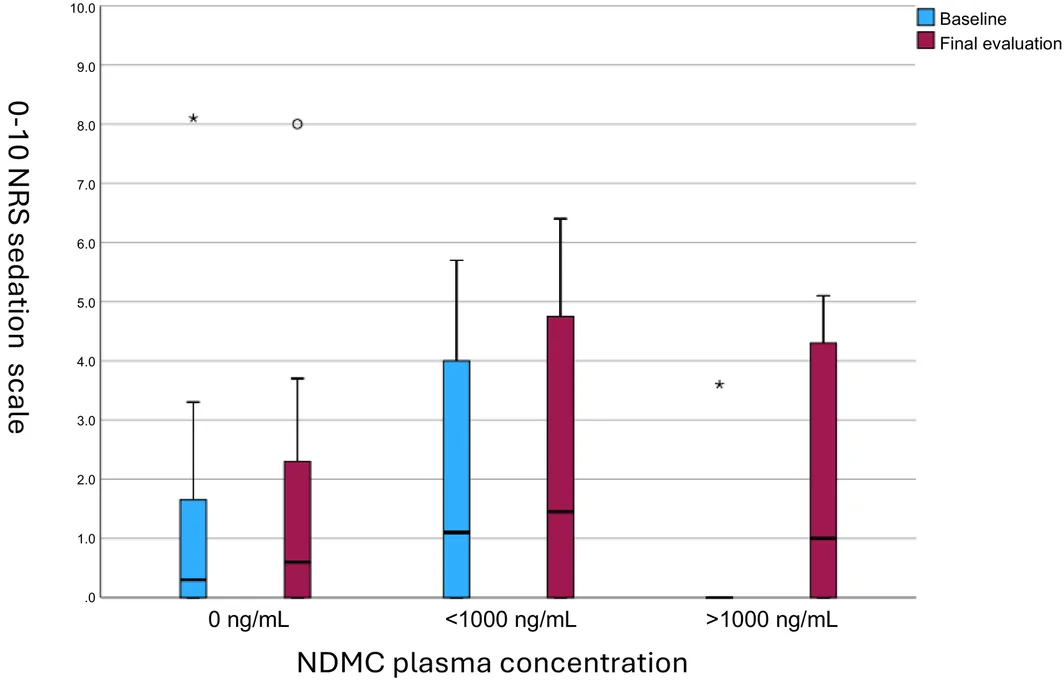
Box plots (min, Q25, median, Q75, max) of Weekly average daily somnolence (WADS) score on a 0 to 10 numerical rating scale (NRS) (mean +/− SD) at baseline and at final evaluation according to NDMC plasma concentrations. Post hoc analysis; final evaluation took place after 4 weeks of treatment maintenance.

**TABLE 5:** Difference in the weekly average of daily somnolence (wads) score measured on a numerating rating scale (NRS) between baseline and final evaluation (4 weeks of treatment maintenance) according to NDMC plasma concentrations; post hoc analysis.

	NDMC conc. = 0 ng/mL, *n* = 11		NDMC conc. < 1000 ng/mL, *n* = 13		NDMC conc. ≥ 1000 ng/mL, *n* = 6		
	Mean	SD	Mean	SD	Mean	SD	Linear reg. *p*
Change in NRS somnolence score	0.24	1.40	0.47	0.95	1.30	1.65	0.12

Table 6 reports the treatment emergent adverse events (TEAE). Seventy percent of the patients reported a TEAE during the study; the incidence varied across treatment groups without any particular trend.

**TABLE 6:** Treatment emergent adverse events.

		Placebo group (*n* = 13)	NDMC 40 mg (*n* = 6)	NDMC 60 mg (*n* = 6)	NDMC 120 mg (*n* = 8)
Total number of AEs, *n*		25	21	15	14
Patients with any AEs *n* (%)		8 (61.5)	6 (100.0)	3 (50.0)	6 (75.0)
Serious AEs, *n* (%)		0	0	0	2 (14.3)
AEs graded severe, *n* (%)		0	2 (9.5)	0	1 (7.1)
Discontinuation due to AEs *n* (%)		0	0	0	1 (7.1)
Incidence of AEs per group *n* (%)		No event	No event	No event	No event
Infections and infestations	Post‐viral fatigue syndrome	0	3 (14.3)	0	0
	COVID‐19	0	0	0	2 (14.3)
Immune system disorders	**Anaphylatic reaction** ^a^	0	0	0	**1 (7.1)**
Psychiatric disorders	Depression	0	0	2 (13.3) [2]	0
	Tension	0	0	2 (13.3) [1]	0
	Depressed mood	2 (8.0) [2]	0	0	0
	Irritability	2 (8.0) [2]	0	0	0
Nervous system disorders	Anosmia	0	1 (4.8)	0	0
	Balance disorder	0	0	1 (6.7) [1]	0
	Dizziness	0	0	0	1 (7.1) [1]
	Headache	1 (4.0)	1 (4.8)	0	0
	Medication overuse headache	0	0	1 (6.7) [1]	0
	Neuralgia	0	3 (14.3)	0	0
	Somnolence	3 (12.0) [3]	3 (14.3) [1]	4 (26.7) [4]	4 (28.6) [4]
Eye disorders	Chalazion	1 (4.0)	0	0	0
Cardiac disorders	**Atrio‐ventricular block complete** ^a^	0	0	0	**1 (7.1)**
Vascular disorders	Hot flush	0	1 (4.8)	0	0
	Ecchymosis	0	1 (4.8)	0	0
Respiratory, thoracic and mediastinal disorders	Productive cough	0	1 (4.8)	0	0
	Cough	0	1 (4.8)	0	0
	Sinus pain	0	1 (4.8)	0	0
Gastrointestinal disorders	Constipation	0	2 (9.5)	0	0
	Anal incontinence	0	0	3 (20.0) [1]	0
	Dyspepsia	2 (8.0) [2]	0	0	0
	Abdominal pain upper	1 (4.0) [1]	0	0	0
	Anal haemorrhage	1 (4.0)	0	0	0
Skin and subcutaneous tissue disorders	Rash erythematous	3 (12.0)	0	0	0
	Urticaria	0	0	0	2 (14.3) [1]
Musculoskeletal and connective tissue disorders	Myalgia	0	1 (4.8)	0	0
	Ligament sprain	0	0	0	1 (7.1)
	Low back pain	0	0	0	1 (7.1)
Renal and urinary disorders	Urinary retention	0	1 (4.8)	0	0
General disorders and administration site	Chills	0	1 (4.8)	0	0
	Influenza like illness	0	0	1 (6.7)	0
	Vaccination site pain	0	0	1 (6.7)	0
	Fatigue	7 (28.0) [7]	0	0	0
	Oedema peripheral	1 (4.0)	0	0	0
Investigations	Weight increased	0	0	0	1 (7.1) [1]
Injury, poisoning and procedural complications	Animal bite	1 (4.0)	0	0	0

*Note:* Bold: severe reactions.

^a^ Serious adverse event in [], number of AEs considered as possibly related to study drug by the investigator.

Two serious adverse events were reported in the NDMC 120 mg group (anaphylactic reaction and complete atrio‐ventricular block) but both were considered as unrelated to the administration of the study drug. Three AEs were graded as severe, 2 occurring in the NDMC 40 mg group (constipation and post‐viral fatigue) and one occurring in the 120 mg group (somnolence), which led to the discontinuation of this patient. Cases of emergent somnolence were observed in all treatment groups, but the frequency was higher in the NDMC groups compared to placebo. Otherwise, AEs were randomly distributed without suggesting a particular pattern of adverse drug reactions associated with NDMC.

### 3.4 Pharmacokinetics

Figure 4 presents the box plots of the NDMC plasma concentrations at steady state (*C*_ss_) in the four treatment groups. The mean NDMC concentrations were 464, 708 and 1292 ng/mL in the NDMC 40 mg, 60 mg and 120 mg, respectively. The concentrations increased linearly with the received doses of NDMC; the correlation between concentrations and doses was 0.92 (*p* = 0.001).

**FIGURE 4:**
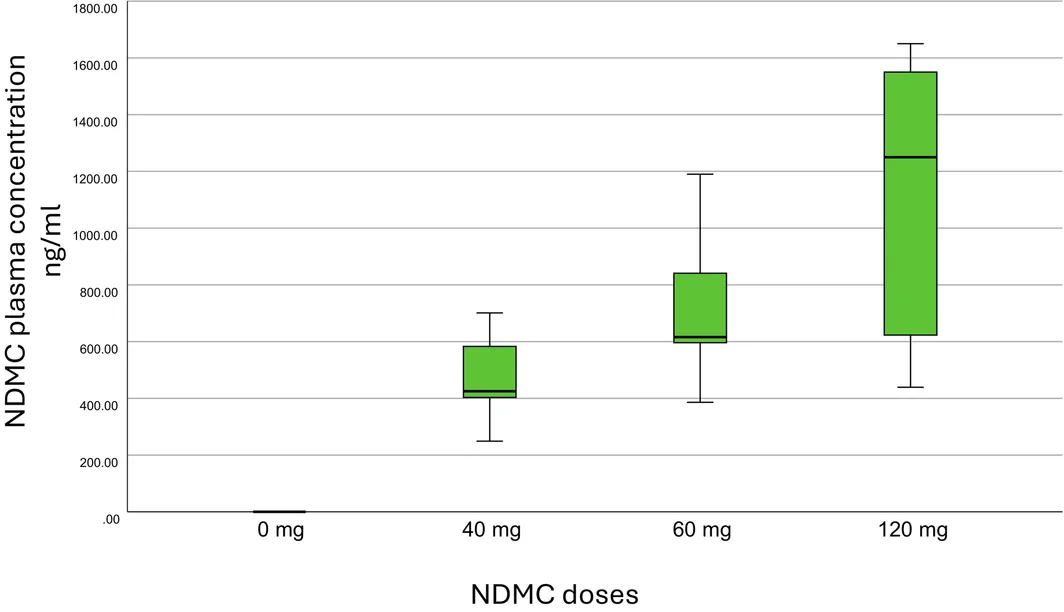
Box plots (min, Q25, median, Q75, max) of NDMC plasma concentrations according to the doses.

## 4 Discussion

The primary objective of this study was to evaluate the efficacy and safety of N‐desmethyl‐clobazam (NDMC) in patients with refractory neuropathic pain.

Conducted during the SARS‐CoV‐2 pandemic, the study was substantially impacted by recruitment constraints. Despite amendments to the protocol aimed at broadening eligibility, as well as an exceptional extension of the initial timeline granted by the funding agency (Innosuisse), recruitment remained insufficient. Consequently, the project was terminated early in accordance with the funding agency's rules. An interim analysis, which was methodologically consistent with the framework of a Phase IIa study, was then performed after one third of the planned recruitment for Cohort 3. The study being clearly underpowered, these data are therefore to be considered exploratory pharmacokinetic and pharmacodynamic findings.

In 30 patients with chronic neuropathic pain, of three to 8 years duration, mainly due to radiculopathy and small fibre polyneuropathy, a mean 2‐points reduction in NRS pain scores was observed from baseline to final assessment. A non‐significant trend towards greater improvement was seen in NDMC‐treated patients, namely, after 4 weeks of maintenance treatment.

In our previous study in healthy volunteers, NDMC exhibited poor absorption following a single oral dose (Matthey et al. 2020). In addition, NDMC undergoes hepatic metabolism predominantly mediated by cytochrome P450 2 C19 (CYP2C19), a genetically polymorphic enzyme. Given the substantial interindividual variability in CYP2C19 activity and the poor absorption, interindividual variability in the disposition of NDMC may therefore be anticipated. This is the reason why we analysed the data according to measured steady‐state plasma concentration of NDMC, with a cut‐off of 1000 ng/mL. This threshold was selected based on our experimental data on the antihyperalgesic effect of NDMC in mice, as well as previous clinical observations in patients with frequent complex partial and/or generalised tonic–clonic seizures (Haigh et al. 1987; Ralvenius et al. 2016).

Stratification by plasma NDMC concentration (< vs. > 1000 ng/mL) showed a consistent pattern: greater pain reduction and an almost twofold higher responder rate (> 30% pain reduction) in the high‐exposure group. The odds ratio for response was 12.5 (95% CI: 1.1–143.5; *p* = 0.04).

Although most comparisons did not reach statistical significance, these converging results suggest a potential efficacy signal, with an estimated NNT of 2.6 for responder status at higher concentrations.

This estimate falls within the range, which was constantly reported in the literature for α2δ‐ligands and antidepressants in neuropathic pain (Armstrong et al. 2007; Lunn et al. 2014; Wiffen et al. 2017) and reviewed in a recent meta‐analysis (NNT: 4.6 (95% CI 3.2–7.7) for tricyclic antidepressants, 7.4 (95% CI 5.6–10.9) for serotonin and noradrenalin reuptake inhibitors and 8.9 (95% CI 7.4–11.1) for α2δ‐ligands (Soliman et al. 2025)).

Improvements in global impression of change, as assessed by both patients and investigators and several qualitof life domains, including mental health, bodily pain, and general health, further supported this trend, with statistically significant effects observed in the ≥ 1000 ng/mL NDMC group.

NDMC showed an expected favourable safety profile with no serious adverse events and good overall tolerability. Adverse event rates were similar across groups, except for somnolence, which occurred approximately twice as often in the NDMC 60 mg and 120 mg groups, consistent with NDMC mechanism of action and relative selectivity. Although being twice as frequent in the NDMC groups, it should be noted that, at most, sedation did not exceed 2.5 points on a 10‐points NRS, which remains clearly in a low and acceptable range from a clinical standpoint.

The pharmacokinetics of NDMC was linear across the dose range, and accumulation following repeated once‐daily administration for 4 weeks enabled effective concentrations to be achieved in all patients, except one who received 120 mg, as expected in our Phase I results (Matthey et al. 2020). One hundred and twenty mg per day is therefore likely to be a target dose.

Based on these preliminary findings, further investigation into the efficacy of NDMC in NP patients is warranted for several reasons.

Neuropathic pain affects approximately 10% of the general population (Attal and Bouhassira 2015). However, currently approved treatments provide only modest benefit: they reduce pain by about 25%–40% on average, and clinically meaningful relief with monotherapy is achieved in at most 40%–60% of patients, partly due to partial efficacy and dose‐limiting adverse effects (Derry et al. 2019; Finnerup et al. 2015; Gilron and Dickenson 2014; Sultan et al. 2008). This finding highlights the need for a multimodal approach targeting the various mechanisms involved in neuropathic pain (Zeng et al. 2025).

Although diminished GABAergic inhibition is a key phenomenon in NP, it has not been targeted before as an analgesic strategy in humans. The many promising compounds available for animal use to modulate this pathway (Zeilhofer et al. 2012) failed early in clinical development because they lost their GABA A receptor α2 over α1 sub‐unit selectivity in humans, leading to unwanted sedation and limiting a translational approach.

In the broader sense, another factor contributing to the failure to bring preclinical compounds to market is poor pharmacokinetics (Harrison 2016).

Both our Phase I study findings (Besson et al. 2015; Matthey et al. 2020) and these preliminary results in NP patients highlight that NDMC has a valuable selectivity profile to support the feasibility of exploring GABAergic modulation in NP patients, potentially showing substantial analgesia with acceptable sedation. This is further reinforced by its well‐established safety record spanning over 40 years, as it is the active metabolite of clobazam. Moreover, pharmacokinetics were proven linear and a dose of 120 mg one time a day generated required steady‐state concentrations.

In addition to conventional small‐molecule therapies, such as peripheral sodium channel blockers (Alsaloum et al. 2020), emerging strategies for pain management include gene therapy or stem cell therapy (Zeng et al. 2025). In the meantime, while these more complex therapeutic approaches are being developed, NDMC remains a simpler option that is already well advanced in its clinical development.

Finally, a major limitation associated with the chronic administration of analgesics is the emergence of tolerance and physical dependence. Preclinical studies have demonstrated that these liabilities, previously reported with non‐selective benzodiazepines, are likely mediated by their interaction with α1‐containing GABA receptors (Tan et al. 2010). In this regard, NDMC may present a favourable pharmacological profile, as it exhibits reduced activity at α1‐containing GABA_A_ receptors in animals, which appeared to be confirmed in humans, given the low level of sedation observed.

Apart from the obvious and significant main limitation of this study, which is the issue of statistical power, certain points are worth highlighting.

In this study, NP was considered as a disease with central sensitisation as a key mechanism, regardless of its origin; consequently, patients with multiple diagnoses have been included.

This has the advantage of clinical relevance, but the more fundamental literature on pharmacological pain management consistently emphasises the need for a more phenotypic and personalised approach (von Hehn et al. 2012).

Furthermore, the efficacy of first‐line treatment, namely antidepressants and α2δ‐ligands, has been tested in homogeneous populations, such as people having painful polyneuropathy, for example (Derry et al. 2019; Finnerup et al. 2015; Wiffen et al. 2017). For further steps of development, and given the mechanism‐based approach, narrowing the aetiology of NP as inclusion criteria would make sense.

Similarly, in this study, NDMC was tested as an add‐on treatment in a third of the included patients. Both clinicians and researchers emphasise the need to test combination therapies, given the limited benefits of monotherapy in NP.

The results of a recent meta‐analysis did not support the conclusion that combinations of α2δ‐ligands‐antidepressants, opioids‐antidepressants or opioids‐α2δ‐ligands were clinically superior, largely due to the heterogeneity of the studies and the often‐small sample sized studies (Balanaser et al. 2023). Still, the authors highlight the potential of accessible and safe combinations in NP and advocate for further research in defined clinical settings using specific drug combinations that safely provide added benefit. NDMC has the required characteristics to form part of such a combination. Specifying in advance whether the combination would involve antidepressant or α2δ‐ligands would enhance the relevance of a future study.

In conclusion, despite being underpowered and stopped early since it was funded exclusively by public funds, this study revealed a consistent exposure–response relationship along with a favourable safety profile for NDMC in patients with NP. These findings support further investigation of NDMC, particularly its role in modulating GABAergic pathways as a therapeutic target for neuropathic pain. The next step would be to proceed with a Phase 2/3 clinical trial.

This objective is especially relevant given that neuropathic pain often shows limited responsiveness to standard pharmacological treatments. Consequently, optimal management is likely to rely on a multimodal approach that targets the various pathways involved in pain pathophysiology.

## Author Contributions

This study was designed by M.B., A.M., Y.D., J.D. and F.C. The experiments were performed by A.M., M.B., A.R. The data were analysed by F.C. and the results were critically examined by all authors. M.B. had a primary role in preparing the manuscript, which was edited by A.M., Y.D., C.S., A.R., J.D. and F.C. All authors have approved the final version of the manuscript and agree to be accountable for all aspects of the work.

## Funding

This work was supported by Swiss innovation agency (Innosuisse) Grant 37848.1 IP‐LS.

## Conflicts of Interest

The authors declare no conflicts of interest.

## Supporting information


**Data S1:** ejp70413‐sup‐0001‐Supinfo.docx.
