# Supplementary material for: The α2‐Subunit Preferring GABA Positive Allosteric Modulator, N‐Desmethyl‐Clobazam in Peripheral Neuropathic Pain Patients: Results of a Phase IIa Randomised Placebo‐Controlled Ascending Dose Study
Source: Eur J Pain. 2026 Oct 16;30(9):e70413. doi: 10.1002/ejp.70413 (PMC13674364; doi:10.1002/ejp.70413)
Supplement: Supplementary file 1 [file EJP-30-0-s001.docx]

Rationale for dose selection:

In the absence of data on any potential anti-hyperalgesic effect of N-desmethly-clobazam (NDMC), the dose rationale was elaborated from our preclinical (Ralvenius et al., 2016) and pharmacokinetic data (Matthey et al., 2020) on NDMC.

Based on the data obtained in HEK293 cells transfected with different subtypes of GABA_A_ receptors, EC_50_ for α2-GABA_A_ receptor was 0.49 µM (with an SD of 0.24) (Ralvenius et al., 2016). Assuming that the number of replicates of this experiment was of 6, we concluded that the therapeutic dose should be in the range of the CI95% of the mean 0.24 to 0.74µM (or 68.4 to 210.9 ng/mL, since NDMC molecular weight is 285 Da). Therefore, to reach similar exposition, NDMC concentrations in the CNS should be between 68.4 ng/mL and 210.9 ng/mL, the mean being 139.7ng/mL.

Furthermore, our preclinical data in mice evidenced that concentration of NDMC ranging from 0.1 to 0.5 µM in CSF would be sufficient to potentiate the GABA_A_ receptors implicated in the nociceptive pathways (Ralvenius et al., 2016).

The CSF/Serum ratio in adults undergoing lumbar puncture was reported 0.1 (Laux and Koeppen, 1984). We assumed that for NDMC the serum/plasma ratio should be close to 1 (Jones and Larsson, 2004; Montenarh et al., 2014).

Studies of the serum protein binding of antiepileptic drugs in a routine clinical setting (Patsalos et al., 2017), demonstrated that NDMC binds with high infinity to plasma proteins (89%). This figure is coherent with the ratio of 0.1 previously described (Laux and Koeppen, 1984) and translates into 10% of free circulating NDMC.

Therefore, to reach these CNS concentrations, blood concentrations of NDMC should be 10 times higher: 684 ng/mL to 2109 ng/mL respectively, with a mean of 1400 ng/mL**.**

Our pharmacokinetics data indicated that a repeated oral administration on NMDC at the dose of 20mg over 14 lead to steady state blood concentrations (SD) of 209 +/- 22 ng/mL, after 15 days (Matthey et al., 2020). NDMC pharmacokinetics of single doses between 20 and 60mg was linear (Matthey et al., 2020).

A total dose of 120mg should therefore generate plasma concentrations in the range of 1’200ng/ mL, well within the CI95% of the mean (684 to 2109 ng/mL),which also falls in the most conservative therapeutic window for efficacy reported for NDMC in epilepsy (de Leon et al., 2013). This value corresponds approximately to CSF concentrations of 0.4 μM (or 114 ng/mL), which falls in the expected efficacy range according to our *in vitro* preclinical data.

Accordingly, repeated daily administrations of NDMC 40mg should have generated plasma concentrations of approximately 400 ng/mL and 40ng/mL (0.13 μM) in the CSF. All doses that were used in the study could have therefore been sufficient to generate a response in NP patients.

References

Jones, A.W., Larsson, H. (2004). Distribution of diazepam and nordiazepam between plasma and whole blood and the influence of hematocrit. *Ther Drug Monit* 26, 380–385.

Laux, G., Koeppen, D. (1984). Serum and cerebrospinal fluid concentration of clobazam and N-desmethylclobazam. *Int J Clin Pharmacol Ther Toxicol* 22, 355–359.

de Leon, J., Spina, E., Diaz, F.J. (2013). Clobazam therapeutic drug monitoring: a comprehensive review of the literature with proposals to improve future studies. *Ther Drug Monit* 35, 30–47.

Matthey, A., Daali, Y., Curtin, F., Poncet, A., Desmeules, J., Besson, M. (2020). GABAergic modulation of secondary hyperalgesia: A randomized controlled 4-way crossover trial with the α2-subunit preferring GABA positive allosteric modulator, N-desmethyl-clobazam in healthy volunteers. *European Journal of Pain* 24, 1094–1106.

Montenarh, D., Hopf, M., Maurer, H.H., Schmidt, P., Ewald, A.H. (2014). Detection and quantification of benzodiazepines and Z-drugs in human whole blood, plasma, and serum samples as part of a comprehensive multi-analyte LC-MS/MS approach. *Anal Bioanal Chem* 406, 803–818.

Patsalos, P.N., Zugman, M., Lake, C., James, A., Ratnaraj, N., Sander, J.W. (2017). Serum protein binding of 25 antiepileptic drugs in a routine clinical setting: A comparison of free non-protein-bound concentrations. *Epilepsia* 58, 1234–1243.

Ralvenius, W.T., Acuña, M.A., Benke, D., Matthey, A., Daali, Y., Rudolph, U., Desmeules, J., Zeilhofer, H.U., Besson, M. (2016). The clobazam metabolite N-desmethyl clobazam is an α2 preferring benzodiazepine with an improved therapeutic window for antihyperalgesia. *Neuropharmacology* 109, 366–375.
